# A Comprehensive Review on Stereotactic Arrhythmia Radioablation (STAR): Pioneering a New Era in Arrhythmia Management

**DOI:** 10.7759/cureus.70601

**Published:** 2024-10-01

**Authors:** Varun N Thawkar, Karuna Taksande

**Affiliations:** 1 Anaesthesiology, Jawaharlal Nehru Medical College, Datta Meghe Institute of Higher Education & Research, Wardha, IND

**Keywords:** arrhythmias, catheter ablation, imaging techniques, patient outcomes, stereotactic arrhythmia radioablation, treatment innovations

## Abstract

Stereotactic arrhythmia radioablation (STAR) is an innovative treatment modality that leverages advanced imaging techniques and focused radiation to target arrhythmogenic foci within the heart, offering a non-invasive alternative to traditional catheter ablation methods. Arrhythmias, characterized by irregular heartbeats, pose significant health risks, including heart failure and stroke, particularly among older adults. Traditional management approaches, such as pharmacological therapies and catheter ablation, often face efficacy, safety, and recurrence limitations. STAR addresses these challenges by utilizing high-precision stereotactic technology to deliver targeted radiation, minimizing damage to surrounding tissues while maximizing therapeutic effects. This review explores the mechanisms, clinical indications, procedural techniques, and outcomes associated with STAR. Recent clinical studies demonstrate that STAR provides comparable efficacy to catheter ablation, with a favorable safety profile, making it a promising option for patients who have not responded to conventional treatments. Integrating STAR into arrhythmia management protocols may enhance patient outcomes and reduce the burden of recurrent arrhythmias. As research in this field advances, STAR holds the potential to reshape the landscape of arrhythmia treatment, providing new hope for patients suffering from these complex conditions. This review aims to elucidate the significance of STAR in modern arrhythmia management and to encourage further exploration and clinical application of this pioneering technique.

## Introduction and background

Arrhythmias are irregular heart rhythms, ranging from benign to life-threatening disturbances. These result from disruptions in the electrical signals that control heartbeats, causing symptoms such as palpitations, dizziness, syncope, and, in severe cases, sudden cardiac arrest [1]. The prevalence of arrhythmias, especially atrial fibrillation (AF) and ventricular tachycardia (VT), represents a significant public health concern, contributing to increased morbidity, mortality, and substantial healthcare costs. As the population ages and the incidence of these disorders rises, the need for effective management strategies has become increasingly critical [2]. Historically, the management of arrhythmias has centered around pharmacological treatments, including antiarrhythmic drugs, anticoagulants, and beta-blockers. While these medications can effectively control symptoms and prevent complications, they are often associated with side effects and limitations [3]. For patients with refractory arrhythmias, catheter ablation has emerged as a viable alternative. This minimally invasive procedure uses thermal energy, such as radiofrequency (RF) or cryoablation, to destroy abnormal cardiac tissue and restore normal heart rhythm. Although catheter ablation is effective, it carries risks such as vascular complications and the potential for arrhythmia recurrence [3].

Stereotactic arrhythmia radioablation (STAR) represents a novel treatment for arrhythmias, utilizing advancements in imaging and radiation delivery technologies. STAR uses high doses of focused radiation to precisely target arrhythmogenic foci, aiming to disrupt abnormal electrical pathways responsible for arrhythmias. This technique offers promise for patients who are not candidates for traditional ablation or those who have not responded to prior interventions [4]. The management of arrhythmias has evolved significantly over the past century. Early treatments were primarily limited to lifestyle modifications and symptom management. The mid-20th century saw the introduction of antiarrhythmic drugs, which marked a turning point by providing patients with more targeted therapeutic options. However, the limitations of pharmacotherapy, particularly for refractory arrhythmias, spurred the development of more invasive treatments [5].

Catheter ablation, introduced in the 1980s, revolutionized the treatment of arrhythmias. Early procedures employed RF energy to ablate abnormal cardiac tissue, establishing a foundation for future innovations. Since then, advancements in mapping technologies and energy delivery systems have improved the safety and efficacy of ablation techniques. Variants like cryoablation have expanded the available options, allowing clinicians to tailor treatments to individual patient anatomy and the specific arrhythmia type [6]. Stereotactic radiotherapy, initially developed for oncology, has gained interest in cardiology. These stereotactic techniques deliver high doses of radiation with pinpoint precision, minimizing damage to surrounding healthy tissue. This technological breakthrough has opened new pathways for treating cardiac arrhythmias, especially in patients unsuitable for catheter-based interventions. The emergence of STAR marks the culmination of these technological advancements, promising a new era in arrhythmia management [7]. This review aims to provide a comprehensive overview of STAR, examining its mechanisms, clinical applications, and outcomes. By synthesizing current research and clinical experiences, this review seeks to showcase the potential of STAR as a transformative treatment for arrhythmias, paving the way for future advancements in the field.

## Review

Mechanism of action

STAR is a cutting-edge, non-invasive technique that uses highly focused radiation beams to target arrhythmogenic foci within the heart. This innovative approach creates localized damage in the myocardium, leading to transmural fibrosis, which interrupts the abnormal electrical pathways responsible for arrhythmias [4]. STAR’s precise stereotactic delivery allows high radiation doses to be administered while minimizing exposure to surrounding healthy tissues. This feature makes STAR particularly beneficial for patients suffering from recurrent VT who have not responded to traditional catheter ablation, offering a novel and promising alternative treatment [4]. When comparing STAR to traditional catheter ablation, several important distinctions arise. Traditional catheter ablation uses RF energy delivered through catheters inserted into the heart via blood vessels. This method requires direct contact with myocardial tissue to create lesions that isolate arrhythmogenic foci [8]. In contrast, STAR is non-invasive, as it does not require direct tissue contact. Additionally, catheter ablation may face challenges reaching deeper myocardial substrates, leading to potential VT recurrences. STAR addresses this limitation by using external radiation capable of penetrating deeper into the heart tissue, which enhances the likelihood of successful outcomes in treating arrhythmias [8]. The success of STAR heavily depends on advanced imaging technologies that ensure precise localization of arrhythmogenic foci. Techniques such as three-dimensional (3D) electroanatomical mapping (EAM) and cross-sectional imaging, including computed tomography (CT) and magnetic resonance imaging (MRI), are used to define the specific regions requiring treatment [9]. This imaging data is integrated into specialized radiation treatment planning software, optimizing the targeting process. Collaboration between electrophysiologists and radiation oncologists is essential to accurately identify and treat the appropriate targets, maximizing treatment success and minimizing potential complications [9].

Clinical indications

STAR is primarily indicated for treating specific arrhythmias, particularly those resistant to conventional therapies [10]. The main arrhythmias STAR targets include VT and ventricular fibrillation (VF). VT, especially recurrent monomorphic VT in patients with structural heart disease, is a central focus of this therapy. VF, known for its life-threatening nature and potential for electrical storms, is another significant target of STAR. However, AF is not typically treated with STAR, as this technique concentrates more on ventricular arrhythmias [10]. Patient selection for STAR involves several critical criteria. Ideal candidates are often those who have experienced unsuccessful catheter ablation or have contraindications to the traditional approach. Patients diagnosed with either ischemic or non-ischemic cardiomyopathy are also considered suitable [11]. Additionally, patients suffering from recurrent monomorphic VT - defined as more than three episodes within the prior three months - are prime candidates, especially when on optimal doses of antiarrhythmic medications as recommended by practitioners. Patients experiencing electrical storms are also strong candidates for consideration. Pre-procedural evaluations typically include comprehensive cardiac imaging (such as CT or MRI), 3D EAM, and electrocardiogram assessments to ensure an accurate and complete assessment before STAR treatment [11]. While STAR offers significant advantages, several contraindications and risks must be considered when selecting patients for the procedure. Pregnancy or breastfeeding is a major contraindication due to the potential risks to the fetus or infant. Patients with a life expectancy of less than six months are also typically excluded from STAR treatment. Additionally, conditions with temporary or genetic causes for VT may render STAR less effective. Patients who remain eligible for catheter ablation may not qualify for STAR [12]. Patients with New York Heart Association Class IV heart failure may also be excluded due to the severity of their condition. Furthermore, polymorphic VT and VF are generally not treated with STAR because of differing underlying mechanisms. Other complications, such as implantable cardioverter-defibrillator (ICD) malfunction and prior chest irradiation, may also contraindicate treatment due to the increased risk of complications [12]. Clinical indications for STAR are summarized in Table 1.

**Table 1:** Clinical indications for stereotactic arrhythmia radioablation (STAR)

Clinical Indication	Description	Patient Population	Evidence Level
Refractory ventricular tachycardia (VT) [13]	For patients with VT that is unresponsive to antiarrhythmic drugs and catheter ablation.	Patients with structural heart disease, post-MI, or with ischemic cardiomyopathy.	High (based on case series and clinical trials)
Atrial fibrillation (AF) [14]	An emerging indication for patients where catheter ablation is not effective or feasible.	Patients with persistent or paroxysmal AF.	Moderate (limited studies available)
Scar-related VT [15]	For VT arising from myocardial scarring, especially in patients with a history of infarction.	Patients with previous myocardial infarction and cardiomyopathy.	High (multiple case series)
Post-cardiac surgery VT [16]	VT occurs after cardiac surgeries such as valve replacements or coronary artery bypasses.	Post-operative cardiac surgery patients.	Moderate (case reports and small studies)
Idiopathic VT [17]	VT without structural heart disease, often originating from the outflow tract.	Younger patients with structurally normal hearts.	Moderate (few case studies)
Refractory atrial flutter [18]	For atrial flutter, it cannot be managed with conventional ablation techniques.	Patients with resistant or recurrent atrial flutter.	Low (limited case reports)
Complex congenital heart disease with arrhythmias [19]	Arrhythmias associated with complex congenital heart anomalies, resistant to other treatments.	Patients with complex congenital heart disease.	Low (case series and expert opinions)

Procedural technique

STAR is a highly complex, multidisciplinary procedure used to treat refractory VT and VF. The procedure requires detailed planning, execution, and post-procedure care to ensure optimal outcomes [20]. The pre-procedure phase begins with comprehensive imaging and mapping to identify the VT substrate. An electrophysiologist typically conducts EAM, which may be based on prior ablation procedures or non-invasive mapping systems. Anatomical scar imaging is also crucial during this phase and can be achieved using various modalities, including echocardiography, CT, MRI, or positron emission tomography (PET) [21]. Once the VT substrate is mapped, the next step involves target volume (TV) delineation. This entails transferring the identified VT substrate onto a contrast-enhanced cardiac CT scan, where radiation oncologists define the TV for treatment planning. This step is vital for delivering effective radiation doses while minimizing collateral damage to surrounding tissues [22]. Another critical aspect of pre-procedure planning is simulating the procedure. This simulation accounts for respiratory and cardiac motion during radiation delivery, ensuring the treatment plan accommodates these movements and accurately targets the arrhythmogenic substrate [23]. Careful patient positioning is essential during the procedure to optimize radiation delivery while maintaining patient comfort and stability throughout the treatment. STAR typically delivers radiation doses between 20 and 30 Gy, depending on the clinical scenario and the target’s characteristics. Lower doses may help reprogram cardiac conduction, while higher doses are associated with inducing scar formation in the targeted area [24]. Radiation delivery requires precise coordination among the multidisciplinary team, which includes electrophysiologists, radiation oncologists, and medical physicists. This collaboration ensures accurate targeting and minimizes risks associated with radiation exposure [25]. Post-procedure, patients require immediate care and close monitoring for any acute side effects or complications related to radiation exposure. Healthcare providers continuously assess vital signs and monitor for potential arrhythmias or other cardiac issues that may arise after the treatment. Follow-up imaging studies may be performed to evaluate the effectiveness of the treatment and monitor changes in cardiac structure or function post-ablation [26]. Long-term monitoring is critical for patients who undergo STAR, as it helps track delayed radiation effects on cardiac tissue and assess the long-term efficacy of the procedure in controlling arrhythmias. Collaboration between electrophysiologists, radiation oncologists, and other healthcare professionals is essential for optimizing patient outcomes and addressing any emerging complications or concerns during the recovery phase [27]. The procedural techniques for STAR are outlined in Table 2.

**Table 2:** Procedural technique for stereotactic arrhythmia radioablation (STAR)

Step	Description	Key Considerations	Equipment/Technology Used
Patient selection [28]	Identification of suitable candidates based on clinical indications and comorbidities.	Assessment of arrhythmia type, heart function, and prior treatment history.	Clinical evaluation, ECG, MRI, CT scans.
Pre-procedure imaging [29]	High-resolution imaging to delineate cardiac anatomy and arrhythmogenic foci.	Image fusion techniques may be used to integrate different imaging modalities.	Cardiac MRI, CT scan, PET scan.
Treatment planning [30]	Creating a treatment plan using radiotherapy planning software to target the arrhythmogenic area.	Precise identification of target volume and dose planning.	Radiotherapy planning software (e.g., eclipse, RayStation).
Immobilization [31]	Ensuring patient stability during the procedure to maintain accurate targeting.	Use of immobilization devices to prevent patient movement.	Immobilization devices (e.g., vacuum cushions, molds).
Image guidance [32]	Continuous imaging to track cardiac and respiratory motion during treatment delivery.	Real-time image guidance is crucial for precise targeting.	4D-CT, fluoroscopy, fiducial markers.
Dose delivery [33]	Delivering a focused radiation dose to the arrhythmogenic site while sparing surrounding tissue.	Typically, a single session of high-dose radiation.	Linear accelerator (LINAC), CyberKnife, Gamma Knife.
Post-procedure monitoring [34]	Monitoring for immediate complications and assessing the effectiveness of the procedure.	Follow-up imaging and arrhythmia monitoring.	ECG, Holter monitoring, MRI/CT scans.
Follow-up and evaluation [35]	Regular follow-up to assess long-term outcomes and manage potential delayed side effects.	Ongoing monitoring of arrhythmia recurrence and cardiac function.	ECG, Holter, MRI, echocardiography.

Efficacy and outcomes

STAR is a cutting-edge, non-invasive treatment method for managing refractory VT and VF, particularly in patients with structural heart disease [36]. STAR uses stereotactic radiotherapy to deliver precisely targeted radiation to cardiac tissue that causes arrhythmias, offering an alternative to traditional catheter ablation, which requires direct contact with the heart tissue. The primary indications for STAR include recurrent VT or VF in patients with structural heart disease, especially in cases where conventional treatments, such as catheter ablation and antiarrhythmic drugs, have been unsuccessful [4]. Recent studies have shown promising outcomes for STAR. A meta-analysis revealed a 92% reduction in VT burden six months after treatment, with 85% of patients requiring fewer than two antiarrhythmic drugs [37]. Furthermore, STAR was associated with a low incidence of severe toxicity, with less than 10% of patients experiencing grade 3 toxicity and no reports of grade 4-5 toxicities. This data has contributed to the growing acceptance of STAR among cardiologists, with many recognizing it as a viable option for patients who do not respond to conventional therapies [37]. Despite these benefits, several obstacles hinder the widespread adoption of STAR. Limited access to the necessary technology is a significant barrier, as only about half of the surveyed cardiologists reported having access to STAR at their institutions. Additionally, variability in treatment protocols and practices for target delineation across institutions may impact treatment outcomes [38]. Efforts like the STOPSTORM consortium address these challenges by establishing standardized treatment protocols and quality assurance measures in participating European centers, aiming to harmonize clinical practices and improve patient outcomes through collaborative research [38]. The future of STAR looks promising, especially as ongoing clinical trials continue to assess its efficacy and safety in larger patient populations. While STAR is expected to become a more widely available treatment for managing VT and VF, it will likely remain a secondary treatment option rather than a first-line therapy, based on the current clinical understanding of its use [39].

Safety and complications

STAR is generally regarded as a safe and effective procedure for treating refractory VT and VF, with a low incidence of complications. However, like any medical intervention, STAR carries some risks. The most common complications include acute toxicity, which manifests as mild to moderate symptoms such as fatigue, skin reactions at the radiation site, and transient arrhythmias following the procedure. Late toxicity, although less frequent, can result in fibrosis or scarring of the irradiated cardiac tissue, potentially leading to long-term complications such as impaired cardiac function [20]. Rare but serious complications include myocardial infarction and exacerbation of heart failure. There is ongoing research into the long-term effects of radiation exposure on the heart, particularly the potential risk of radiation-induced malignancies or other radiation-related damage to cardiac structures. According to a meta-analysis involving 61 STAR-treated patients, late-grade three toxicity occurred in only 2% of cases, and no grade 4 or 5 toxicities were reported, underscoring the relative safety of the procedure [20]. Management of complications associated with STAR requires a multidisciplinary approach, with close monitoring during and after the procedure. Acute reactions such as skin irritation or fatigue can typically be managed with symptomatic treatments, including analgesics for pain or topical medications for skin reactions [40]; for more severe complications, such as myocardial infarction or arrhythmias, immediate medical intervention may be required, often involving hospitalization and the implementation of standard cardiac care protocols. Long-term follow-up is critical for monitoring late-onset complications and maintaining cardiac function post-procedure [40]. To minimize risks, several preventive strategies are employed. Patient selection is crucial, excluding individuals with a life expectancy of less than six months or pregnant women. Pre-procedural imaging and mapping help to localize the arrhythmogenic substrate accurately, minimizing unnecessary radiation exposure to healthy tissue. A multidisciplinary team of electrophysiologists, radiation oncologists, and medical physicists collaborates to ensure optimal patient care throughout the treatment process [41]. Standardized treatment protocols and continuous education and training for healthcare professionals involved in STAR procedures are vital for improving safety outcomes and reducing complication rates across different centers [41]. Table 3 highlights the safety and complications associated with STAR.

**Table 3:** Safety and complications associated with stereotactic arrhythmia radioablation (STAR)

Category	Potential Complication	Description	Incidence/Severity	Management/Prevention
Radiation-related [42]	Radiation pneumonitis	Inflammation of lung tissue due to radiation exposure.	Moderate to rare	Corticosteroids, supportive care, dose constraints.
	Esophageal injury	Radiation-induced esophagitis or ulceration, potentially leading to perforation.	Rare	Dose limitation, careful planning, proton pump inhibitors (PPIs).
	Skin toxicity	Radiation-induced skin reactions, ranging from erythema to ulceration.	Low to moderate	Skin care regimen, dose modulation.
	Cardiac tissue damage	Unintended damage to the myocardium, pericardium, or coronary arteries.	Low but potentially severe	Advanced imaging for precise targeting and dose constraints.
Arrhythmia-related [43]	Pro-arrhythmic effects	New or worsening arrhythmias due to tissue irritation or damage.	Rare but possible	Close monitoring, antiarrhythmic medications, and ICD use if needed.
	Heart block	Damage to the conduction system, leading to partial or complete heart block.	Rare	Pacemaker implantation if required.
Pulmonary complications [44]	Pneumothorax	The accumulation of air in the pleural space is potentially caused by radiation or procedural intervention.	Rare	Chest tube placement, conservative management.
	Bronchial fistula	Abnormal connection between bronchial tree and other structures due to tissue damage.	Very rare	Surgical intervention if necessary.
General complications [26]	Fatigue	General fatigue post-procedure due to radiation exposure and stress.	Common	Rest, hydration, supportive care.
	Nausea/vomiting	Possible side effects due to radiation or stress.	Low	Anti-emetics, hydration, supportive care.
	Late toxicities	Potential for late-onset complications, such as fibrosis or secondary malignancies.	Very rare	Long-term monitoring and regular follow-up.

Future directions

The future of STAR holds significant promise, particularly in imaging innovations, expanded treatment indications, and its integration with other therapeutic modalities [36]. One of the most exciting advancements is improving cardiac imaging and targeting techniques. Enhanced imaging modalities, such as high-resolution MRI and PET/CT scans, can substantially increase the accuracy in identifying arrhythmia substrates and delineating treatment targets for STAR [45]. By integrating electroanatomic mapping data from traditional catheter ablation procedures with advanced imaging techniques, clinicians can refine TVs, leading to optimized treatment outcomes. Developing novel cardiac imaging biomarkers may also allow for better predictions of radiation-induced effects, enabling more personalized and effective STAR treatment plans [45]. As STAR continues to evolve, there is potential for expanding its indications beyond refractory VT. STAR may be considered for a broader range of patients, such as those with advanced heart failure and limited life expectancy who are seeking compassionate treatment options when other therapies have failed. Additionally, researchers may explore the feasibility of using STAR to treat other arrhythmias, including AF. With technological advancements, these expansions could greatly increase the applicability of STAR in clinical practice [46]. Another promising future direction for STAR involves its integration with other therapeutic strategies. Combining STAR with antiarrhythmic drugs or catheter ablation as part of a multimodal approach could enhance overall efficacy while reducing recurrence rates. Defining the optimal timing of STAR and other therapies - whether as an upfront treatment or following a failed catheter ablation - will be crucial in establishing its place within the broader treatment algorithm. Moreover, exploring synergistic effects between STAR and novel therapies, such as gene or cell therapies, could pave the way for groundbreaking combination treatments that further improve patient outcomes [47]. Table 4 outlines future directions in STAR.

**Table 4:** Future directions in stereotactic arrhythmia radioablation (STAR)

Future Direction	Description	Potential Impact	Challenges
Integration with advanced imaging [48]	Utilizing advanced imaging modalities like PET-MRI for better identification of arrhythmogenic foci.	Improved targeting accuracy and reduced complications.	High cost, need for specialized equipment and expertise.
Adaptive radiotherapy [49]	Real-time treatment plan adaptation based on cardiac and respiratory motion during delivery.	Enhanced precision and reduced exposure to surrounding tissue.	Technological complexity, real-time imaging limitations.
Personalized treatment planning [50]	Use patient-specific models and AI algorithms to tailor treatment parameters and dose distribution.	Customized treatments lead to improved outcomes.	Data requirements and validation of AI models.
Combined modality approach [51]	Integration of STAR with other therapies such as catheter ablation or antiarrhythmic drugs.	Synergistic effects, improved efficacy in complex cases.	Determining optimal combinations and risk of increased side effects.
Expanded indications [52]	Exploring the use of STAR for other arrhythmias like refractory AF and congenital arrhythmias.	Broader applicability, offering treatment to more patients.	Limited clinical evidence, need for more trials.
Reduction of late toxicities [53]	Developing techniques to minimize long-term side effects such as fibrosis and secondary malignancies.	Improved long-term safety and patient quality of life.	Understanding of long-term radiobiological effects.
Enhanced patient selection criteria [54]	Utilizing biomarkers and genetic profiling to better identify candidates who will benefit from STAR.	Increased efficacy and reduced unnecessary interventions.	Development and validation of reliable biomarkers.
Non-invasive real-time monitoring [55]	Implementation of non-invasive methods for continuous monitoring of arrhythmia recurrence post-STAR.	Early detection of recurrence and improved follow-up care.	Technological advancements require patient compliance.
Cost-effectiveness studies [56]	Studies will be conducted to evaluate the cost-effectiveness of STAR compared to conventional therapies.	Justification for broader clinical adoption.	High cost of implementation, variability in healthcare systems.
Multicenter clinical trials [57]	Large-scale, multicenter trials to validate efficacy and safety across diverse populations.	Robust clinical evidence and standardized protocols.	Coordination, funding, and patient recruitment.

## Conclusions

In conclusion, STAR represents a transformative advancement in managing arrhythmias, offering a promising alternative to traditional treatment modalities. By harnessing the precision of stereotactic imaging and focused radiation, STAR enables clinicians to effectively target and ablate arrhythmogenic foci with minimal collateral damage to surrounding tissues. The evidence emerging from clinical trials indicates that STAR not only holds comparable efficacy to conventional catheter ablation but also offers a favorable safety profile, making it a viable option for patients with limited treatment options. As research continues to expand our understanding of this innovative technique, it is essential to integrate STAR into comprehensive arrhythmia management protocols. By doing so, healthcare professionals can enhance patient outcomes, reduce the burden of recurrent arrhythmias, and ultimately improve the quality of life for those affected by these complex conditions. The future of arrhythmia management is poised for significant change, with STAR at the forefront of this evolution, promising new hope for patients and practitioners alike.
